# Hemodynamic effects of COVID-19 vaccination in hospitalized patients awaiting heart transplantation

**DOI:** 10.1016/j.ahjo.2022.100168

**Published:** 2022-07-05

**Authors:** Rachel E. Ohman, Michael C. DiVita, Meshe Chonde, Stephanie Fraschilla, Ali Nsair, Daniel Cruz, Jeffrey J. Hsu

**Affiliations:** a Department of Medicine, University of California Los Angeles, 757 Westwood Plaza, Los Angeles, CA 90095, USA; b Division of Cardiology, Department of Medicine, NYU Grossman School of Medicine, 530 1st Avenue, Skirball 9U, New York, NY 10016, USA; c Cedars-Sinai Smidt Heart Institute, 127 South San Vicente Boulevard, Suite A6100, Los Angeles, CA 90048, USA; d Division of Cardiology, Department of Medicine, University of California Los Angeles, 100 Medical Plaza, Suite 630 West, Los Angeles, CA 90095, USA; e Division of Cardiology, Department of Medicine, Greater Los Angeles Veteran Affairs Healthcare System, 11301 Wilshire Boulevard, Los Angeles, CA 90073, USA

**Keywords:** COVID-19, SARS-CoV-2, Vaccination, Heart failure, Heart transplantation, Hemodynamics

## Abstract

**Background:**

The hemodynamic effects of pre-transplant vaccination against COVID-19 among heart transplant candidates hospitalized for advanced heart failure remains unknown.

**Methods:**

A retrospective chart review was conducted at a high-volume transplant center from January through December 2021. 22 COVID-19 vaccination events occurred among patients hospitalized for decompensated heart failure while awaiting transplantation. Primary outcomes included inotrope and vasopressor dosages. Secondary outcomes included vital signs, pulmonary artery catheter measurements, diuretic dosages, and renal function. Data were extracted 24 h before through 72 h after vaccination.

**Results:**

One of 22 vaccination events was associated with hemodynamic changes requiring increased inotropic and vasopressor support post-vaccination. In all other cases, transient hemodynamic changes occurred without need for escalated therapy.

**Conclusions:**

COVID-19 vaccination can be administered safely to most critically ill patients with advanced heart failure including those awaiting transplantation. All patients should be monitored closely as some may be susceptible to significant hemodynamic changes.

## 1 Introduction

Although increasing numbers of patients in the United States have been vaccinated against coronavirus disease 2019 (COVID-19), many remain unvaccinated or incompletely vaccinated. While mRNA vaccination against SARS-CoV-2 is well-tolerated in heart transplant recipients [1], [2], immunization in these patients [1], [2], [3] and other solid organ transplant recipients [4], [5] has been associated with impaired immunogenicity. To maximize protection against COVID-19, the American Society of Transplantation and the International Society of Heart and Lung Transplantation recommend vaccination of transplant candidates before organ transplant whenever possible to achieve optimal immunity before the initiation of immunosuppression [6]. However, given the susceptibility of patients with advanced heart failure (HF) to infectious and inflammatory exposures, the impact of potential vaccine-induced inflammatory responses in critically ill patients with decompensated HF may be of concern. We aim to characterize pre-transplant vaccination in this population by examining the immediate impact of COVID-19 vaccination on the hemodynamics of hospitalized patients with HF awaiting heart transplantation.

## 2 Methods

A retrospective chart review at a high-volume transplant center was conducted among all heart transplant recipients from January through December 2021 who were hospitalized and listed, or under evaluation for listing, for transplant at the time of COVID-19 vaccination. Data were extracted at predetermined time points 24 h before and continued through 72 h after vaccination. Primary outcomes included changes in inotrope or vasopressor infusion rates. The maximal vasoactive inotrope score (VIS) was calculated manually using the formula suggested by Gaies et al [7] and validated in adult populations [8], [9] (VIS = dopamine dose (μg/kg/min) + dobutamine dose (μg/kg/min) + 100 × epinephrine dose (μg/kg/min) + 10 × milrinone dose (μg/kg/min) + 10,000 × vasopressin dose (U/kg/min) + 100 × norepinephrine dose (μg/kg/min)). Secondary outcomes included vital signs, hemodynamic measurements from pulmonary artery catheters, total daily dosage of loop diuretics, thiazide diuretic use, urine output, arrhythmias, and serologic measures of renal function and blood cell counts. Fever was defined as temperature ≥38 °C and hypothermia as temperature ≤35 °C. Any post-vaccination changes in mean arterial pressure (MAP), pulmonary artery pressures (PAP), and cardiac index (CI) were quantified by percentage change from measurements in the preceding 24 h. Changes were quantified as mild if ≤20 % in MAP, ≤30 % in CI, or ≤30 % in systolic, diastolic, or mean PAP. Changes were quantified as non-sustained if lasting for ≤12 h. Quantification of daily loop diuresis was calculated manually via determination of furosemide dose equivalents. Augmentation with thiazide was categorized as a binary variable indicating presence or absence of use. Serologic values were abstracted from daily morning bloodwork. Acute kidney injury was categorized by KDIGO criteria [10]. Arrhythmias were detected from cardiac monitor records and progress notes. Univariate analyses were performed due to the small sample size and exploratory study nature.

## 3 Results

Of the 63 patients who underwent orthotopic heart transplant from January through December 2021, 45 were vaccinated against COVID-19 with at least one dose before undergoing transplantation. Of the remaining 18 patients, 13 were vaccinated against COVID-19 after heart transplantation. No reasons were documented for the delay in vaccination. The other five patients remain unvaccinated against COVID19. Three of the five have not been vaccinated due to patient hesitancy. The other two patients have no documented reasons for their lack of vaccination.

**Supplemental Fig. 1:**
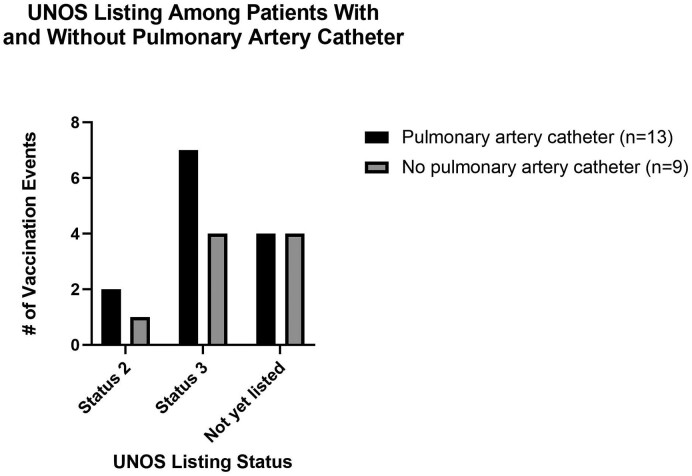
**UNOS Listing Among Patients with and without a Pulmonary Artery Catheter.** Of the 17 patients who received a COVID-19 vaccine dose while hospitalized in the cardiac critical care unit before heart transplantation, ten patients representing 13 vaccination events had a pulmonary artery catheter in place at the time of immunization. Seven patients representing nine vaccination events did not have a pulmonary artery catheter in place at the time of vaccination. The UNOS listing status is depicted for each vaccination event, with the black bars representing events that occurred in patients with a pulmonary artery catheter and gray bars representing events that occurred in patients without a pulmonary artery catheter.

**Supplemental Fig. 2:**
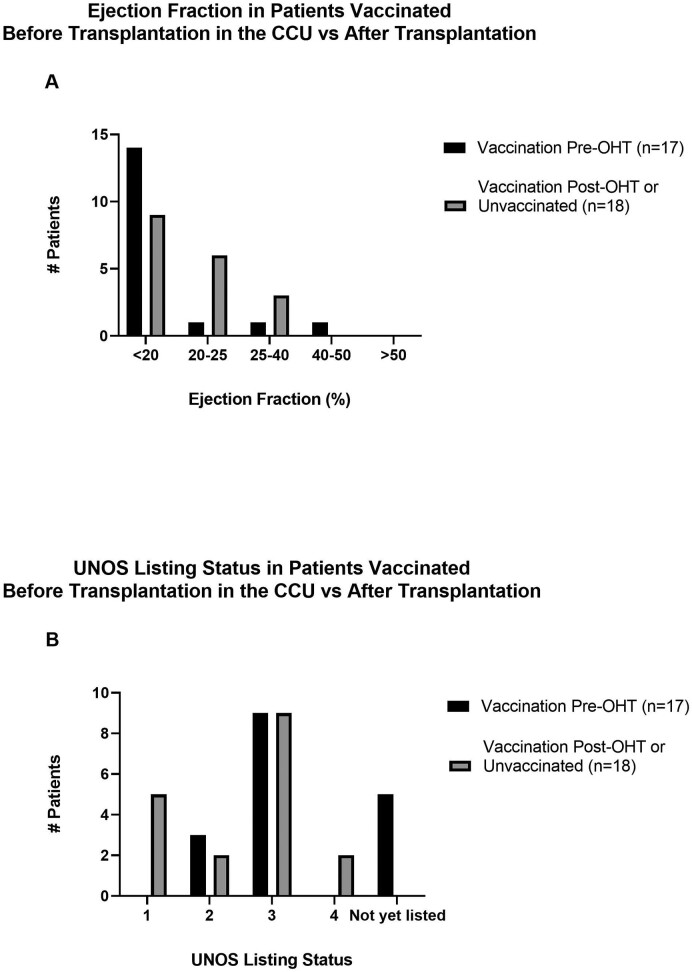
**Ejection Fraction and UNOS Listing Status in Patients Vaccinated Before Transplantation in the Cardiac Critical Care Unit (CCU) vs After Transplantation.** A comparison of (A) left ventricular ejection fraction and (B) UNOS listing status at the time of vaccination is provided for the 17 patients vaccinated against COVID-19 before transplantation while hospitalized in the cardiac critical care unit, represented by black bars, versus the 18 patients either vaccinated after transplantation (n=13) or not at all (n=5), represented by gray bars.

Of the 45 patients vaccinated against COVID-19 before heart transplantation, 17 received a COVID-19 vaccine dose while hospitalized in the cardiac critical care unit awaiting heart transplantation. As some patients received two vaccine doses while hospitalized, a total of 22 vaccination events were identified from the 17 patients meeting inclusion criteria. Five of these 17 patients were on mechanical circulatory support, but none required changes to device settings during the study period. Ten patients representing 13 vaccination events had a pulmonary artery catheter at the time of immunization. A summary of patient characteristics is detailed in Table 1. A comparison of the United Network for Organ Sharing (UNOS) listing status among patients vaccinated with and without a pulmonary artery catheter is included in Supplemental Fig. 1. Comparisons of the left ventricular ejection fraction and UNOS listing status between patients vaccinated before transplantation and those vaccinated after transplantation or not at all can be found in Supplemental Fig. 2. There is a notable difference in UNOS listing status: five patients vaccinated after heart transplantation or not at all were listed as UNOS status 1 at the time of transplantation, while none of the patients included in this study were listed as status 1 at the time of vaccination.

### 3.1 Primary outcomes

**Fig. 1:**
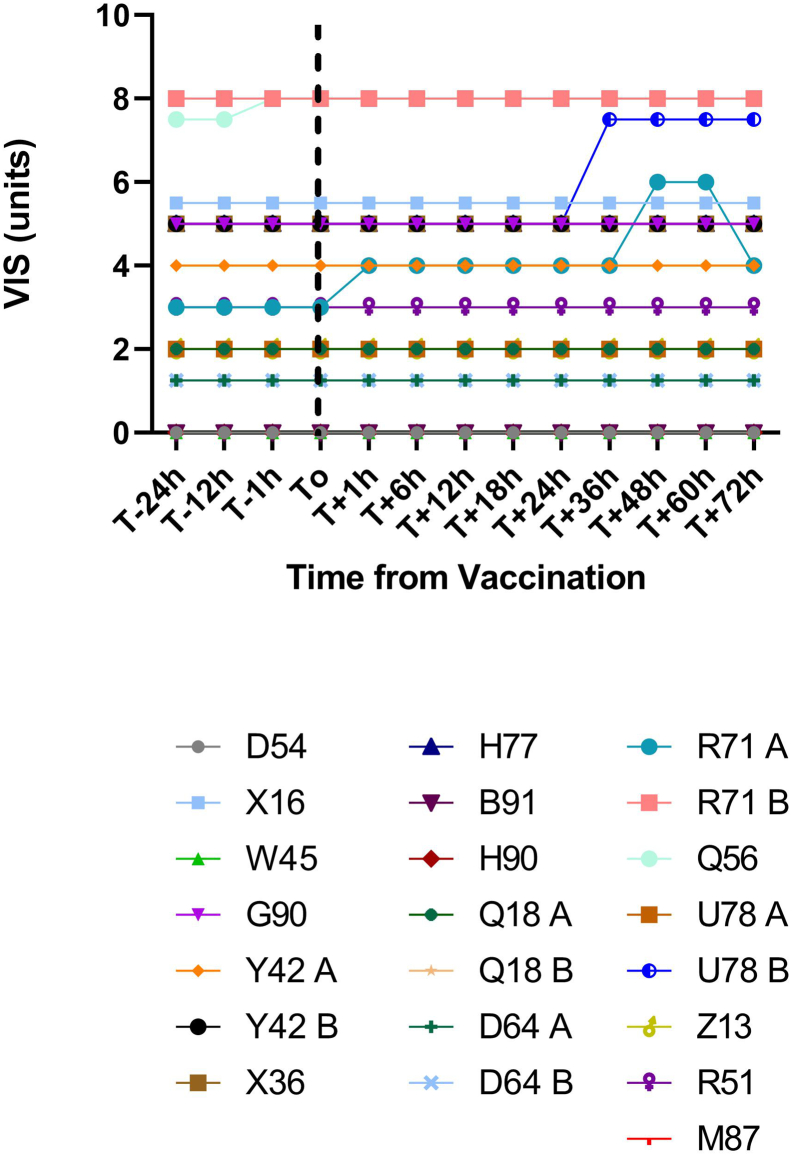
Vasoactive inotrope score (VIS) after COVID-19 vaccination. VIS was calculated at designated time points 24 h before and through 72 h after COVID-19 vaccination for each vaccination event. Each patient was assigned a randomly generated alphanumeric code. Separate vaccination events for the same patient were labeled as “A” and “B.” Vaccination event B in patient U78 was notable for an increase in VIS from 5 to 7.5 (coinciding with initiation of dobutamine 2.5 μg/kg/min) 36 h after immunization. Vaccination event A in patient R71 was notable for an increase in VIS from 3 to 4 (coinciding with an increase in milrinone by 0.1 μg/kg/min) 1 h after vaccination, and an increase in VIS from 4 to 6 (coinciding with initiation of dopamine at 2 μg/kg/min) 48 h after immunization. No other vaccination events were associated with changes in VIS.

Of the 22 vaccination events, the majority (20/22, 91 %) did not require changes in inotropic or vasopressor support within 72 h of immunization (Fig. 1). Two cases required inotrope or vasopressor adjustments. In the first case (R71 A), the VIS increased from 3 to 4 as milrinone was increased for elevated pulmonary artery pressures present before vaccination. At 48 h after vaccination the VIS increased from 4 to 6 due to the initiation of dopamine for pre-existing oliguric cardiorenal injury without hemodynamic changes. Dopamine was discontinued by 72 h after vaccination. This event occurred after the patient received the first dose of the Pfizer-BioNTech vaccine.

**Table 1:** Demographic and clinical characteristics of study participants.

Category	Patients (n = 17)
Age, years	52 (45–60)
Sex	
Male	11 (64.7 %)
Female	6 (35.3 %)
Race/ethnicity	
Hispanic/Latinx	6 (35.3 %)
Unspecified	5 (29.4 %)
Mexican/Mexican American/Chicano	1 (5.9 %)
White	5 (29.4 %)
Black	2 (11.8 %)
Asian and Pacific Islander	2 (11.8 %)
Samoan	1 (5.9 %)
Filipino	1 (5.9 %)
Other	2 (11.8 %)
Employment status	
Unemployed/unknown	12 (70.6 %)
Employed	3 (17.6 %)
Retired	1 (5.9 %)
Disabled	1 (5.9 %)
Cardiomyopathy etiology	
Non-ischemic	13 (76.5 %)
Ischemic	3 (17.6 %)
Mixed	1 (5.9 %)
Non-ischemic cardiomyopathy types	
Unknown	6 (46.2 %)
Chagas	2 (15.4 %)
Familial	1 (7.7 %)
Viral	1 (7.7 %)
Cytotoxic	1 (7.7 %)
Valvular	1 (7.7 %)
Peri-partum	1 (7.7 %)
Ejection fraction at time of vaccination (%)	
<20	14 (82.4 %)
20–25	1 (5.9 %)
25–40	1 (5.9 %)
40–50	1 (5.9 %)
>50	0
NYHA class on admission	
II	3 (17.6 %)
III	9 (52.9 %)
IV	5 (29.4 %)
Mechanical circulatory support	
Percutaneous LVAD	3 (17.6 %)
Durable LVAD	2 (11.8 %)
IABP	2 (11.8 %)
Renal function	
Hemodialysis	2 (11.8 %)
Transplant type	
Heart	14 (82.4 %)
Heart and kidney	3 (17.6 %)


Category	Vaccination events (n = 22)
COVID-19 vaccine type	
Pfizer-BioNTech	11 (50.0 %)
Moderna	7 (31.8 %)
Johnson & Johnson	4 (18.2 %)
COVID-19 vaccine dose	
Dose #1	15 (68.2 %)
Dose #2	6 (27.3 %)
Dose #3	1 (4.5 %)
Transplant listing status at vaccination	
Not yet listed	8 (36.4 %)
Status ≥4	0 (0 %)
Status 3	11 (50.0 %)
Status 2	3 (13.6 %)
S tatus 1	0 (0 %)

Demographic and clinical data are displayed as number of patients or number of vaccination events (percentage of total sample), except for the age of the study participants which is presented as the median value (interquartile range). Race and ethnicity are documented as recorded in the electronic medical record, with ethnicity sub-groups included if available. Note that two patients were designated with the race of “Other” without further specification. For patients on mechanical circulatory support, there were two patients with a durable left ventricular assist device, two patients with a percutaneous left ventricular assist device and concurrent intra-aortic balloon pump, and one patient with a percutaneous left ventricular assist device alone. Abbreviations: COVID-19, coronavirus disease 2019; IABP, intra-aortic balloon pump; LVAD, left ventricular assist device; NYHA, New York Heart Association.

**Fig. 2:**
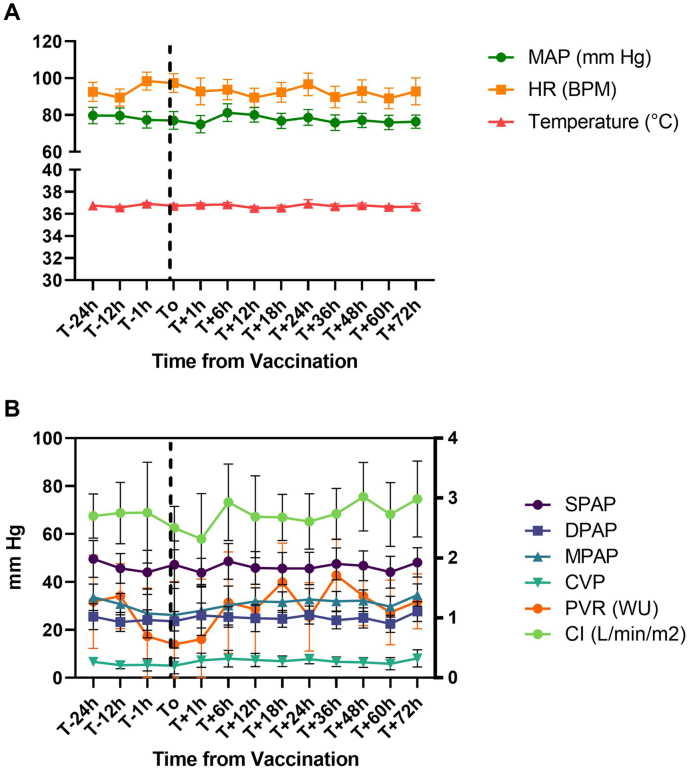
Mean change in hemodynamic parameters after COVID-19 vaccination. A) Average mean arterial pressure (MAP), heart rate (HR), and temperature 24 h before and through 72 h after vaccination among all vaccination events (n = 22, bars represent 95 % confidence intervals). B) Average systolic pulmonary artery pressure (SPAP), diastolic pulmonary artery pressure (DPAP), mean pulmonary artery pressure (MPAP), central venous pressure (CVP), pulmonary vascular resistance (PVR), and cardiac index (CI) 24 h before and through 72 h after vaccination among all vaccination events with a pulmonary artery catheter (n = 13, bars represent 95 % confidence intervals). Pulmonary vascular resistance data are scaled according to the right axis in Woods Units (WU). Cardiac index data are scaled according to the right y axis in L/min/m^2^.

The other case (U78 B) involved a patient started on a second inotrope and new vasopressor for hemodynamic changes. 24 h after receiving the second dose of the Moderna vaccine, the patient developed a fever to 38.1 °C that defervesced with acetaminophen without recurrence. Over the next day the patient developed worsening CI and elevated PAP, prompting the initiation of continuous dobutamine with continuation of pre-existing continuous milrinone, totaling a VIS increase from 5 to 7.5. The patient was started on a continuous bumetanide infusion to escalate diuresis. Despite these efforts, the patient developed worsening hypotension 72 h after vaccination. Shortly thereafter the patient received empiric antibiotics and vasopressin (not depicted in Fig. 2 as beyond the study period) for suspected mixed shock and underwent urgent placement of an intra-aortic balloon pump before achieving stabilized hemodynamics.

### 3.2 Secondary outcomes

In the other 20 vaccination events and the case of R71 A described above (21/22, 95 %), changes occurred in MAP, PAP, or CI in the first 72 h after immunization without the need for escalated inotropes or vasopressors (Fig. 2). All changes were mild except for one case in which the patient experienced a 37 % increase in mean PAP without changes in CI, with improvement after routine hemodialysis. In the majority of cases (17/22, 77 %), changes were non-sustained. In only four cases (18 %) did patients experience changes lasting up to 72 h, all of which improved without medication adjustments. As demonstrated in Fig. 2, these reflect post-capillary changes in PAP with pulmonary vascular resistance remaining below three Woods Units throughout the study period.

In four cases (18 %), patients required increased loop diuretic dosage, and in two cases (9 %) patients required thiazide diuretic initiation. In four cases (18 %), patients experienced acute kidney injury. One patient experienced an asymptomatic tachyarrhythmia that responded to oral beta blocker up-titration without the use of intravenous medication. These and other secondary outcomes are detailed in Supplemental Table 1. Of note, there was no documented evidence of post-vaccination myocarditis in any patient. In 11 cases (50 %), patients endorsed subjective symptoms after vaccination that resolved with supportive care (Supplemental Table 2). The most common symptoms reported were myalgias (18 %), fatigue (14 %), and nausea (14 %).

## 4 Discussion

In this retrospective examination of early hemodynamic effects of COVID-19 vaccination among patients hospitalized for advanced HF while awaiting heart transplantation, one of 22 vaccination events was followed by hemodynamic changes requiring increased inotropic and vasopressor support. The patient had tolerated the first Pfizer-BioNTech vaccination dose during an earlier hospitalization without difficulty, although the patient's cardiac function had worsened by the time of the second immunization. Whether or not the patient's decompensation was related to vaccination remains unknown, but warrants consideration.

Reassuringly, most patients underwent vaccination without complications. In 21 of 22 vaccination events, patients experienced transient decreases in blood pressure, increases in PAP, or decreases in cardiac function that resolved without adjustment to vasoactive medications. The majority of these changes were mild (91 %) and non-sustained (77 %). 64 % of vaccination cases had stable diuretic requirements and renal function.

The current study was a retrospective chart review at a single high-volume heart transplant center. Sample size was limited by the available population of patients meeting inclusion criteria. Seven of the 17 patients did not have pulmonary artery catheters at the time of vaccination. Importantly, the study may not have included patients deemed too hemodynamically unstable for vaccination, supported by the lack of patients listed status 1 in the study population. This may have biased results toward immunization effects in patients considered by cardiac care teams to be sufficiently stable for vaccination.

## 5 Conclusions

These data suggest that COVID-19 vaccination can be administered safely to most critically ill patients with advanced HF awaiting heart transplantation. However, all patients should be monitored closely after immunization as some may be susceptible to significant hemodynamic changes. These findings should inform prospective multicenter investigations to help safely close gaps in COVID-19 vaccination in this vulnerable population.

The following are the supplementary data related to this article.

**Supplemental Table 1. Incidence of Clinical Events After Vaccination**
Incidence of changes in mean arterial pressure, changes in temperature, acute or acute on chronic kidney injury, leukocytosis or neutropenia, and arrhythmia events after COVID-19 vaccination among all vaccination events (n=22). Incidence of changes in pulmonary artery pressures and changes in cardiac function are separately reported for the vaccination events that occurred among patients with a pulmonary artery catheter (n=13). Incidence values are reported as number of vaccination events (percentage of sample).
^a^All episodes of leukocytosis were acute on chronic.
^b^Both instances of neutropenia were in patients with prior episodes of intermittent neutropenia.
^c^In this case, the patient experienced hemodynamically stable atrial flutter for four minutes and then again for ten minutes, with subsequent up-titration of oral beta-blocker dosage without the administration of any intravenous medications.
**Supplemental Table 2. Incidence of Clinical Symptoms After Vaccination**
Incidence of clinical symptoms reported by patients after COVID-19 vaccination among all vaccination events (n=22). Incidence values are reported as number of vaccination events (percentage of sample). Image 1

## Funding

This research did not receive any specific grant from funding agencies in the public, commercial, or not-for-profit sectors.

## Declaration of competing interest

The authors declare that they have no known competing financial interests or personal relationships that could have appeared to influence the work reported in this paper.
