# Supplementary material for: Hemodynamic effects of COVID-19 vaccination in hospitalized patients awaiting heart transplantation
Source: Am Heart J Plus. 2022 Jul 5;18:100168. doi: 10.1016/j.ahjo.2022.100168 (PMC9252923; doi:10.1016/j.ahjo.2022.100168)
Supplement: Supplementary file 1 — Supplemental Table 1. Incidence of Clinical Events After Vaccination Incidence of changes in mean arterial pressure, changes in temperature, acute or acute on chronic kidney injury, leukocytosis or neutropenia, and arrhythmia events after COVID-19 vaccination among all vaccination events (n=22). Incidence of changes in pulmonary artery pressures and changes in cardiac function are separately reported for the vaccination events that occurred among patients with a pulmonary artery catheter (n=13). Incidence values are reported as number of vaccination events (percentage of sample). aAll episodes of leukocytosis were acute on chronic. bBoth instances of neutropenia were in patients with prior episodes of intermittent neutropenia. cIn this case, the patient experienced hemodynamically stable atrial flutter for four minutes and then again for ten minutes, with subsequent up-titration of oral beta-blocker dosage without the administration of any intravenous medications. Supplemental Table 2. Incidence of Clinical Symptoms After Vaccination Incidence of clinical symptoms reported by patients after COVID-19 vaccination among all vaccination events (n=22). Incidence values are reported as number of vaccination events (percentage of sample). [file mmc3.docx]

**Supplemental Table 1**

| **Event Incidence, No. (%)** | **Vaccination Events (n=22)** |
| --- | --- |
| Decrease in mean arterial pressure | 18 (82) |
| Hyperthermia | 2 (9.1) |
| Hypothermia | 1 (4.5) |
| Acute kidney injury | 6 (27.3) |
| Leukocytosis^a^ | 4 (18.2) |
| Neutropenia^b^ | 1 (4.5) |
| Arrhythmia^c^ | 1 (4.5) |
| **Event Incidence, No. (%)** | **Vaccination Events (n=13)** |
| Increase in pulmonary artery pressures | 11 (84.6) |
| Decrease in cardiac index | 10 (76.9) |

**Supplemental Table 1. Incidence of Clinical Events After Vaccination**

Incidence of changes in mean arterial pressure, changes in temperature, acute or acute on chronic kidney injury, leukocytosis or neutropenia, and arrhythmia events after COVID-19 vaccination among all vaccination events (n=22). Incidence of changes in pulmonary artery pressures and changes in cardiac function are separately reported for the vaccination events that occurred among patients with a pulmonary artery catheter (n=13). Incidence values are reported as number of vaccination events (percentage of sample).

^a^All episodes of leukocytosis were acute on chronic.

^b^Both instances of neutropenia were in patients with prior episodes of intermittent neutropenia.

^c^In this case, the patient experienced hemodynamically stable atrial flutter for four minutes and then again for ten minutes, with subsequent up-titration of oral beta-blocker dosage without the administration of any intravenous medications.

**Supplemental Table 2**

| **Event, No. (%)** | **Vaccination Events (n=22)** |
| --- | --- |
| Myalgias | 4 (18.2) |
| Fatigue | 3 (13.6) |
| Nausea +/- emesis | 3 (13.6) |
| Chills | 2 (9.1) |
| Headache | 2 (9.1) |
| Chest pain | 2 (9.1) |
| Dizziness | 1 (4.5) |

**Supplemental Table 2. Incidence of Clinical Symptoms After Vaccination**

Incidence of clinical symptoms reported by patients after COVID-19 vaccination among all vaccination events (n=22). Incidence values are reported as number of vaccination events (percentage of sample).
